# Fibroblast growth factor 21 alleviates acute pancreatitis via activation of the Sirt1‐autophagy signalling pathway

**DOI:** 10.1111/jcmm.15190

**Published:** 2020-03-31

**Authors:** Qiongzhen Chen, Jinmeng Li, Junfeng Ma, Xiaoning Yang, Ming Ni, Yali Zhang, Xiaokun Li, Zhuofeng Lin, Fanghua Gong

**Affiliations:** ^1^ College of Life and Environmental Science Wenzhou University Wenzhou China; ^2^ School of Pharmacy Wenzhou Medical University Wenzhou China

**Keywords:** acute pancreatitis, autophagy signalling pathway, fibroblast growth factor 21, inflammation, Sirt1

## Abstract

Fibroblast growth factor 21 (FGF21), a metabolic hormone with pleiotropic effects on glucose and lipid metabolism and insulin sensitivity, alleviates the process of acute pancreatitis (AP). However, its mechanism remains elusive. The pathological and physiological characteristics of FGF21 are observed in both patients with AP and cerulein‐induced AP models, and the mechanisms of FGF21 in response to AP are investigated by evaluating the impact of autophagy in FGF21‐treated mice and cultured pancreatic cells. Circulating levels of FGF21 significantly increase in both AP patients and cerulein‐induced AP mice, which is accompanied by the change of pathology in pancreatic injury. Replenishment of FGF21 distinctly reverses cerulein‐induced pancreatic injury and improves cerulein‐induced autophagy damage in vivo and in vitro. Mechanically, FGF21 acts on pancreatic acinar cells to up‐regulate Sirtuin‐1 (Sirt1) expression, which in turn repairs impaired autophagy and removes damaged organs. In addition, blockage of Sirt1 accelerates cerulein‐induced pancreatic injury and weakens the regulative effect in FGF21‐activated autophagy in mice. These results showed that FGF21 protects against cerulein‐induced AP by activation of Sirtuin‐1‐autophagy axis.

## 1 INTRODUCTION

Acute pancreatitis (AP) is one of the most common acute abdomen diseases with a case fatality rate of 20%. It is primarily caused by excessive alcohol consumption, bile duct obstruction or drug allergy. Its pathological features are the abnormal expression of prozymogen granules, excessive activation of digestive enzymes in acinar cells, pancreatic injury, fibrosis and inflammatory reactions.^1^, ^2^, ^3^, ^4^ AP is often accompanied by severe complications and multiple organ dysfunction, carrying a high mortality rate.^5^, ^6^

Fibroblast growth factor 21 (FGF21) was found to be a metabolic regulator that specifically acts on liver, islet and adipose tissue.^7^, ^8^ Despite the fact that FGF21 is highly expressed in the pancreas, little is known about its role in pancreatitis. It was found that the expression of FGF21 increased during the development of AP, and FGF21 may play an important role in pancreatitis.^9^, ^10^, ^11^ Moreover, FGF21 has been found to inhibit pancreatic fibrosis and inflammation in AP.^12^ However, the mechanism of FGF21 regulation in AP remains unclear.

Autophagy is the formation of autophagosomes by phagocytosis of a cell's own cytoplasm, organelles and proteins that need to be degraded, followed by fusion with a lysosome to form an autolysosome that degrades the contents of the autophagosome, so as to satisfy the metabolic needs of the cell itself and the renewal of some organelles.^13^, ^14^ AP causes organelle dysfunction including in lysosomes and mitochondria, resulting in poor autophagy progression/efficiency.^15^, ^16^ In a study of the inhibitory effect of FGF21 on diabetes mellitus, it was found that autophagic damage in cells induced expression of FGF21, thereby inhibiting obesity and insulin resistance‐induced diabetes.^17^, ^18^ Nevertheless, whether the protective effect of FGF21 against AP is related to autophagy remains unclear. Sirtuin‐1 (Sirt1) is a representative member of the histone deacetylase family. It reduces the activity of transcription factors and down‐regulates inflammatory transcription genes by deacetylating histones, nuclear factor kappa B (NF‐kB) and activator protein 1 (AP‐1) in vivo.^19^, ^20^ It has been found that Sirt1 regulates autophagy and promotes phagocytosis of abnormal organelles and proteins.^21^, ^22^ In addition, Sirt1 also plays an important role in the regulation of diabetic cardiomyopathy and lipid metabolism by FGF21.^23^, ^24^ However, whether Sirt1 mediates the protective effect of FGF21 in response to cerulein‐induced AP keep further investigated. In this study, we investigated the molecular mechanism of FGF21 against cerulein‐induced AP. Our results demonstrate that FGF21 alleviates cerulein‐induced AP by activation of Sirt1/autophagy signalling axis, which in turn repairs damaged mitochondria and lysosomes, inhibits the abnormal expression of prozymogen granules and inflammatory response and finally improves cerulein‐induced acute pancreatic injury.

## 2 METHODS

### 2.1 Serum of AP clinical patients

We collected serum from patients with AP in the First Affiliated Hospital of Wenzhou Medical University. Depending on diagnostic results, AP patients were divided into two subgroups, severe and mild AP, with 30 patients in each group. Mild and severe AP clinical diagnostic criteria were as follows: mild acute pancreatitis (MAP) with the clinical manifestations of AP (acute, persistent abdominal pain) and biochemical changes (serum amylase three times higher than normal ceiling, elevated inflammatory factor expression, etc), and no organ dysfunction or local complications. Severe acute pancreatitis (SAP) with the clinical manifestations of AP and biochemical changes, and one of the following: local complications (pancreatic necrosis, pseudocyst, pancreatic abscess); Organ failure; Ranson score ≥3; APACHE‐II score of 8 or higher; CT grades were D and E.

### 2.2 Animals and reagents

Healthy male C57BL/6 mice, 6‐8 weeks old, were purchased from Nanjing Model Biology Research Institute. All procedures complied with the standards for the care and use of animal subjects, as stated in the Guide for the Care and Use of Laboratory Animals, and were approved by the Committee on Animal Health and Care of Wenzhou Medical University. They were raised in separate cages within SPF‐level barriers in the Animal Laboratory Center of Wenzhou Medical University. Room temperature was maintained at 23 ± 1°C, humidity at 60% and a 12‐hour light/12‐hour dark cycle. The mice were fed adaptively for 1 week before the experiment. The mice were fasted 12 hours before modelling but were not forbidden to drink. All animals were fed and used in accordance with the guidelines of the National Institutes of Health of China. Cerulein was purchased from Sigma‐Aldrich. Recombinant human FGF21 protein was provided by the Key Laboratory of Biotechnology Pharmaceutical Engineering, Wenzhou Medical University. The recombinant lentivirus of small interfering RNA targeting Sirt1 (Sirt1‐RNAi lentivirus) and the control lentivirus (Control‐RNAi‐lentivirus) were designed and synthesized by Genechem Co. Ltd. Antibodies against FGF21, LC3, Beclin1, P62, LAMP1 and LAMP2 were purchased from Abcam; antibodies against Sirt1, β‐actin and GAPDH were purchased from Cell Signaling Technology. An amylase (AMS) detection kit was purchased from Nanjing Jiancheng Bioengineering Institute. ELISA Kits for IL‐6, TNF‐α, FGF21 and Sirt1 were purchased from Abcam.

### 2.3 Animal group and model establishment of acute pancreatitis

Mice were randomly divided into control group (Control), AP model group (AP), FGF21 treatment group (AP＋FGF21) and AP model group with FGF21 treatment and Sirt1 inhibition (Sirt1‐RNAi+AP +FGF21), and each group has six samples. The establishment of the AP model in mice with cerulein has been detailed in the literature.^2^, ^3^ Briefly, AP was induced in mice by intraperitoneal injection of 50 μg/kg cerulein seven times, at intervals of 1 hour. The control group was given the normal saline. The mice in the FGF21 treatment group were injected with 1 mg/kg recombinant human FGF21 intraperitoneally and then were intraperitoneal injected with 50 μg/kg cerulein seven times at intervals of 1 hour. In the AP model group with FGF21 treatment and Sirt1 inhibition, each mouse was injected intraperitoneally with Sirt1‐RNAi lentivirus (4 × 10^8^ TU/mouse) for 1 week and then intraperitoneally with 50 μg/kg of cerulein seven times at intervals of 1 hour. We injected 1 mg/kg FGF21 intraperitoneally before cerulein injections.

### 2.4 Determination of AMS, IL‐6, TNF‐α, FGF21 and Sirt1

After anaesthesia, venous blood from each mouse was sampled by cardiac puncture. The supernatants were separated by centrifugation for 15 minutes at 300 *g*/min and stored at −80°C until analysis. The levels of AMS, inflammatory cytokines IL‐6, TNF‐α, FGF21 and Sirt1 were determined according to manufacturers’ protocols.

### 2.5 H&E staining

Pancreatic tissue was harvested and fixed in 10% formalin for more than 48 hours and then embedded in paraffin. The sections of 5‐µm thickness were stained according to the standardized H&E staining procedure. Twelve visual fields were randomly selected under the microscope, and based on Schmidt's scoring criteria, the degree of pancreatic injury was assessed using a 10‐point scoring system by observing the degree of oedema, the number of vacuoles, the width of lobular space, the amount of cell necrosis and inflammatory cytokines.

### 2.6 AR42J cells and Sirt1 lentivirus

AR42J cells were purchased from the Cell Bank of Chinese Academy of Sciences and were cultured with high‐glucose DMEM medium containing 10% FBS, in humid air with 5% CO_2_ at 37°C. An in vitro AP model was established by stimulating AR42J cells with 10^−8^ mol/L cerulein for 2 hours. The effect of FGF21 on the in vitro AP model was investigated by stimulating the AP model cells with FGF21 (200 ng/mL) for 2 hours. The recombinant lentiviruses, small interfering RNA targeting Sirt1 (Sirt1‐RNAi lentivirus) and control lentivirus (Control‐RNAi‐lentivirus), were designed and synthesized by Genechem Co. Ltd.

### 2.7 Western blotting

The total proteins in pancreatic tissues or cells were extracted by reagents and were quantified using a BCA protein assay kit. The proteins were separated by SDS‐PAGE gel electrophoresis and transferred onto PVDF membranes (Bio‐Rad). The membranes were blocked with TBST solution containing 10% (w/v) skimmed milk powder for 1.5 hours at room temperature and then were incubated with FGF21 antibody (1:1000), Sirt1 antibody (1:5000), LC3 antibody (1:2000), Beclin 1 antibody (1:1000), P62 antibody (1:10 000), LAMP1 antibody (1:1000), LAMP2 antibody (1:2000), β‐actin antibody (1:1000) and GAPDH antibody (1:1000) overnight in a shaking bed at 4°C. Subsequently, the membranes were washed three times with TBST and incubated with secondary antibody coupled with horseradish peroxidase at room temperature for 1 hour. Signal of protein was detected using a gel imaging system (Tanon‐5200).

### 2.8 Detection of intracellular structure of pancreatic cells by transmission electron microscopy

Pancreas tissue pieces <1 mm^3^ were harvested and fixed overnight in 2.5% glutaraldehyde phosphate buffer. Then, they were washed twice with 0.1 mol/L PBS for 15 minutes each time, fixed with 1% osmium acid for 1 hour, washed twice with 0.1 mol/L PBS for 15 minutes each time, stained with 2% uranium acetate solution for 30 minutes and then dehydrated with 50%, 70% and 90% alcohol in turn for 15 minutes each; they were dehydrated with 100% alcohol once for 20 minutes and then with 100% acetone twice for 20 minutes each. About 1:1 infiltration:anhydrous acetone and embedding agent were mixed in equal volumes to infiltrate the tissue and shaken for 2 hours. Pure embedding agent was used to infiltrate the tissue and shake for 2 hours. We embedded the tissue with pure embedding agent and then polymerized it in an oven. The temperature and time of polymerization were 37°C for 24 hours, 45°C for 24 hours and 60°C for 48 hours. Final steps included repairing and ultrathin slicing (about 120 nm); 4% uranium acetate staining for 20 minutes; and lead citrate staining for 5 minutes; finally, stained ultrathin sections were placed on a single‐hole copper mesh and photographed using TECNAI 10 transmission electron microscopy.

### 2.9 Data analysis

All analyses were performed using GraphPad Prism 5.0 software. Data are expressed as mean ± SEM. Statistical significance was determined using Student's *t* test (for comparison of two experimental conditions) or analysis of variance (ANOVA) (for comparison of three or more experimental conditions). The linear correlation of two variables was determined using the Pearson test. In all statistical comparisons, a *P* value < .05 indicated a statistically significant difference.

## 3 RESULTS

### 3.1 FGF21 is largely increased in patients with AP and cerulein‐induced AP mice

**Figure 1:**
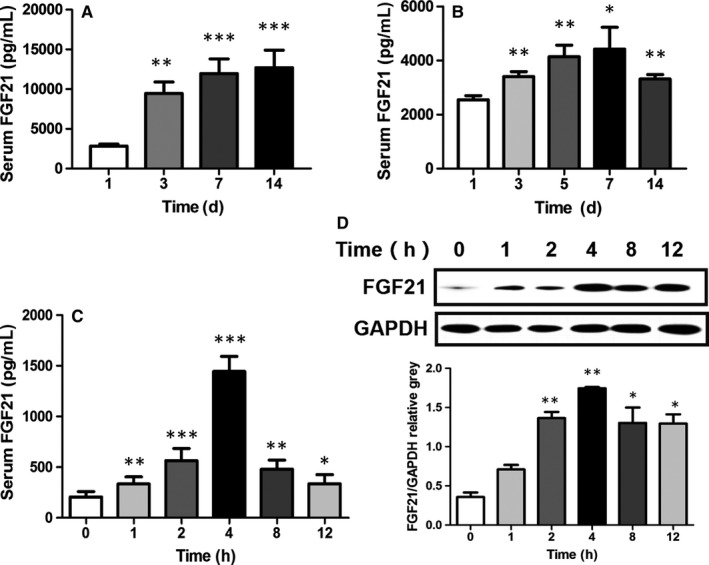
FGF21 is largely increased in patients with AP and cerulein‐induced AP mice. A, Serum FGF21 levels in patients with mild pancreatitis at 1, 3, 7 and 14 d after admission. B, Serum FGF21 levels in patients with severe pancreatitis at 1, 3, 5, 7 and 14 d after admission. C, Changes in serum FGF21 levels in mice at various time points after treated with cerulein. D, The changes of FGF21 levels in pancreatic tissue at various time points determined using Western blot. ***P < .001, **P < .01, *P < .05

Clinical‐ and animal‐based studies have explored the relationship between FGF21 and AP. To further explore the role of FGF21 and AP, we firstly investigated the change of FGF21 in patients with AP and cerulein‐induced AP mice. Consistent with previous study, serum FGF21 levels and pancreatic FGF21 contents were markedly increased in AP patients with a critical condition and a stable condition (Figure 1A,B). Interestingly, circulating FGF21 levels in patients with AP were also strongly associated with the development of pathogenesis of AP (Figure 1A), and serum FGF21 levels in AP patients with critical condition were markedly higher than those in a stable condition (eg: 11 650 ± 3848 pg/mL vs 4421 ± 807 pg/mL at 7 days, *P* < .05). Consistently, circulating FGF21 levels and pancreatic FGF21 contents were also significantly increased in cerulein‐induced AP mice (Figure 1C,D). These results suggest that FGF21 may be involved in the pathogenesis of AP.

### 3.2 Replenishment of FGF21 attenuates cerulein‐induced pancreatic injury

To further clarify the role of increased FGF21 in AP, we next explored whether replenishment of FGF21 could attenuate cerulein‐induced AP in mice. Consistent with previous reports, treatment with cerulein severely caused pancreatic injury, as accompanied by increasing the wet weight of pancreatic tissue, and severely induced the change of pathological morphology in pancreatic tissue of mice, followed by increasing pancreatic oedema, massive vacuoles and cell necrosis. In addition, the Schmidt's score, a marker of pancreatic injury in cerulein‐treated mice, was markedly increased with a time‐dependent manner. On the other hand, administration of cerulein also significantly increased serum AMS levels, as well as the expression of IL‐6 and TNF‐α (Figure S1).

**Figure 2:**
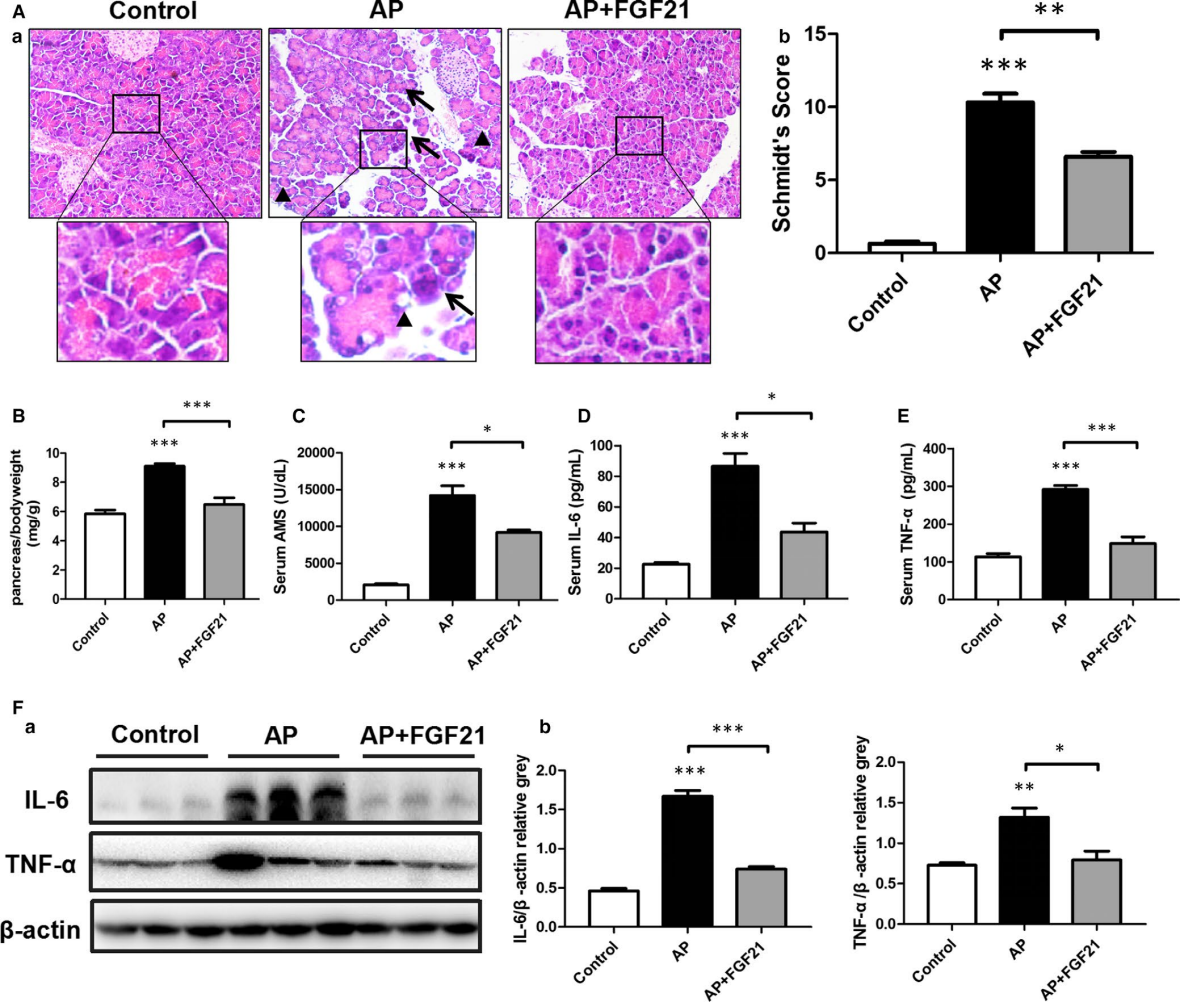
FGF21 alleviates the pathological damage of AP. A (a), H&E staining was used to assess pancreatic injury in mice in various groups (black triangle: tissue vacuoles, black arrow: cell necrosis). (b) The Schmidt's scores of pancreases. B, The changes of wet weight of pancreas in mice after various administrations. C, The changes of serum AMS levels in mice at various administrations. D, E, Changes in IL‐6 and TNF‐α serum levels in mice. F, Changes in IL‐6 and TNF‐α levels in pancreatic tissue of mice determined by Western blot. ***P < .001, **P < .01, *P < .05

Interestingly, these negative effects were strongly attenuated by intraperitoneal injection of recombinant human FGF21 protein. Treatment with FGF21 strongly decreased the ratio of pancreas to bodyweight, the Schmidt's Score, serum AMS levels as well as circulating levels of systemic inflammatory factors IL‐6 and TNF‐α (Figure 2A‐E). Furthermore, replenishment of FGF21 also alleviated the pancreatic contents of inflammatory factors IL‐6 and TNF‐α (Figure 2F).

In light of the animal results that replenishment of FGF21 markedly attenuated cerulein‐induced AP, we further evaluated the protective effect of FGF21 in response to cerulein‐treated pancreatic cells. As expected, treatment of cultured AR42J cells with cerulein largely increased the expression of FGF21 protein in a dosage‐dependent manner, but significantly decreased pancreatic FGF21 contents in a time‐dependent manner (Figure S2A,B). In addition, administration of AR42J cells with recombinant FGF21 protein markedly attenuated cerulein‐induced pancreatic cellular injury, following with reversed cerulein‐induced increase of AMS levels, and the expression of pro‐inflammatory mediators IL‐6 and TNF‐α (Figure S2C‐F). Taken together, these results suggest that FGF21 presents a protective effect in response to cerulein‐induced acute pancreatic injury.

### 3.3 Activation of autophagy is involved in cerulein‐induced acute pancreatic injury in mice and cultured cells

**Figure 3:**
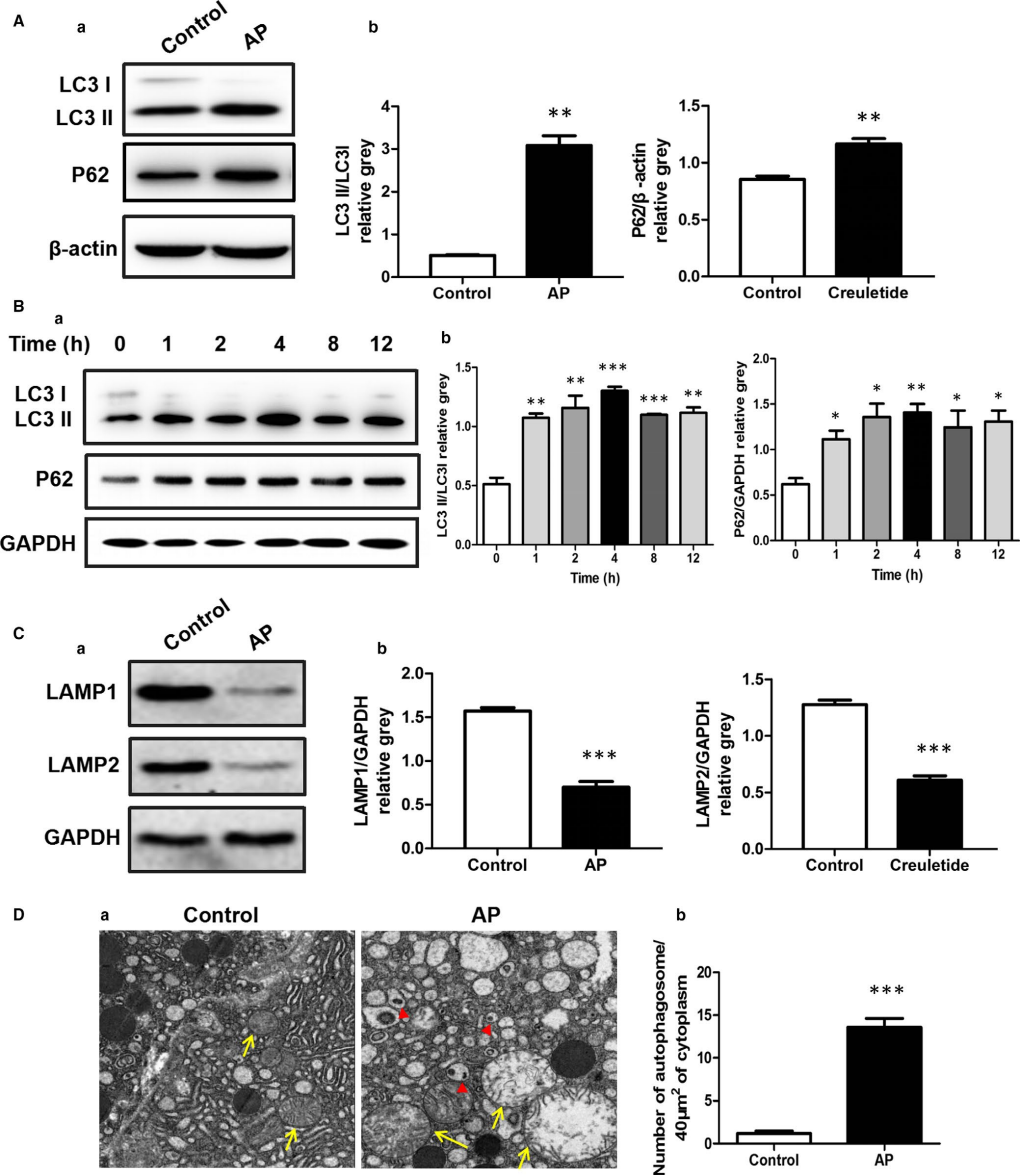
Activation of autophagy is involved in cerulein‐induced acute pancreatic injury in mice and cultured cells. A, Expression level changes of LC3II/I, P62 in AR42J cells treated with 10^−8^ mol/L cerulein for 2 h. B, Expression levels of autophagy‐related proteins LC3II/I, P62 in pancreatic tissue of mice with AP at various time points. C, Expression level changes of lysosomal membrane proteins LAMP1 and LAMP2 in pancreatic tissue. D (a) The changes of organelles and autophagosomes in pancreatic tissue assessed by transmission electron microscopy (yellow arrow: mitochondria, red triangle: autophagosomes). (b) Analysis chart: The number of autophagosomes was counted every 40 µm^2^ (10 fields). ***P < .001, **P < .01, *P < .05

**Figure 4:**
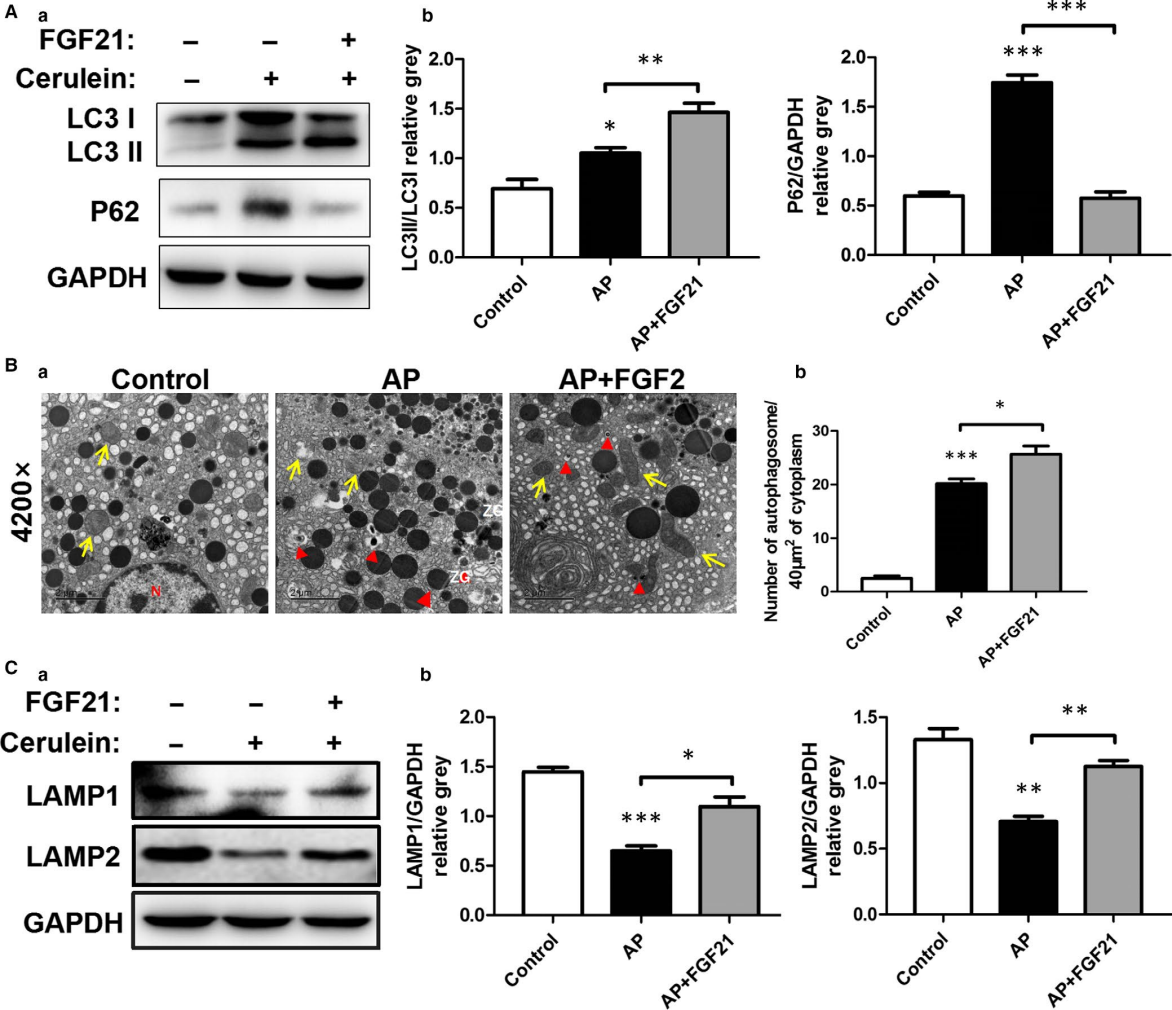
Autophagy medicated the protective effect of FGF21 against cerulein‐induced acute pancreatic injury. A, Expression level changes of LC3II/I, P62 of various treatment groups in pancreatic tissue. B (a), The changes of organelles and autophagosomes in pancreatic tissue assessed by transmission electron microscopy (yellow arrow: mitochondria, red triangle: autophagosomes). (b) Analysis chart: The number of autophagosomes was counted every 40 µm^2^ (10 fields). C, Expression level changes of lysosomal membrane proteins LAMP1 and LAMP2 in pancreatic tissue. ***P < .001, **P < .01, *P < .05

Previous studies have reported that pancreatic autophagy was activated in response to cerulein‐induced pancreatitis.^25^, ^26^, ^27^ Autophagic removal of damaged mitochondria (termed mitophagy) is a well‐established cellular adaptive mechanism to prevent cell damage. Since FGF21 has been shown to potentiate acetaminophen‐induced autophagy in mice, we next investigated whether autophagy would medicate the protective effect of FGF21 against cerulein‐induced acute pancreatic injury. Consistent with previous reports, treatment with cerulein induced formation of autophagosomes and decreased endogenous levels of LC3‐I as well as the expression of pancreatic LAMP1 and LAMP2, two important lysosomal membrane proteins, which play an important role in the integration of autophagosomes and lysosomes, but the degradation of p62 was blocked (Figure 3). Interestingly, these effects were strongly attenuated after treatment with recombinant FGF21 proteins by intraperitoneal injection, as accompanied by the improvement of pathologic status in cerulein‐induced AP mice (Figure 4), suggesting that the protective of FGF21 on cerulein‐induced acute pancreatic injury may be related to the activation of autophagy in mice.

### 3.4 Loss of Sirt1 inhibits activation of autophagy and accelerates cerulein‐induced acute pancreatic injury

**Figure 5:**
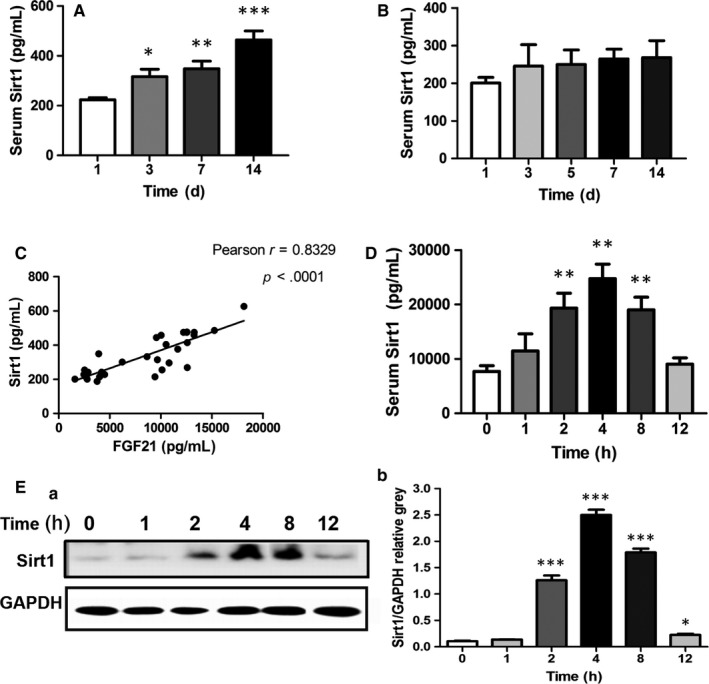
Sirt1 is involved in the pathogenesis of AP. A, Serum Sirt1 levels in patients with mild pancreatitis at 1, 3, 7 and 14 d after admission. B, Serum Sirt1 levels in patients with severe pancreatitis at 1, 3, 5, 7 and 14 d after admission. C, Pearson's correlation test for serum FGF21 and Sirt1 levels in patients with mild acute pancreatitis. D, Changes in serum Sirt1 levels in mice at various time points. E, The changes of Sirt1 levels in pancreatic tissue at various time points determined using Western blot. ***P < .001, **P < .01, *P < .05

Sirtuin 1 (Sirt1), a longevity gene related to many diseases associated with dysregulation of metabolism, is a nicotinamide adenine dinucleotide (NAD+)‐dependent protein deacetylase and master metabolic regulator, which has been confirmed to regulate the activation of autophagy. To further explore the role of autophagy in cerulein‐induced AP, we next investigated whether Sirt1 is involved in AP by regulating the autophagy in cerulein‐treated mice. Interestingly, serum Sirt‐1 levels in AP patients with a severe condition were significantly increased during the pathological processes of AP (Figure 5A,B). In addition, circulating Sirt1 levels were also positively correlated with the change of serum FGF21 levels in patients with AP (Figure 5C). In line with the change of Sirt1 in AP patients, circulating Sirt1 levels and pancreatic Sirt1 contents were also obviously elevated in cerulein‐induced AP mice (Figure 5D,E), suggesting that Sirt1 is related to the pathological processes of AP.

**Figure 6:**
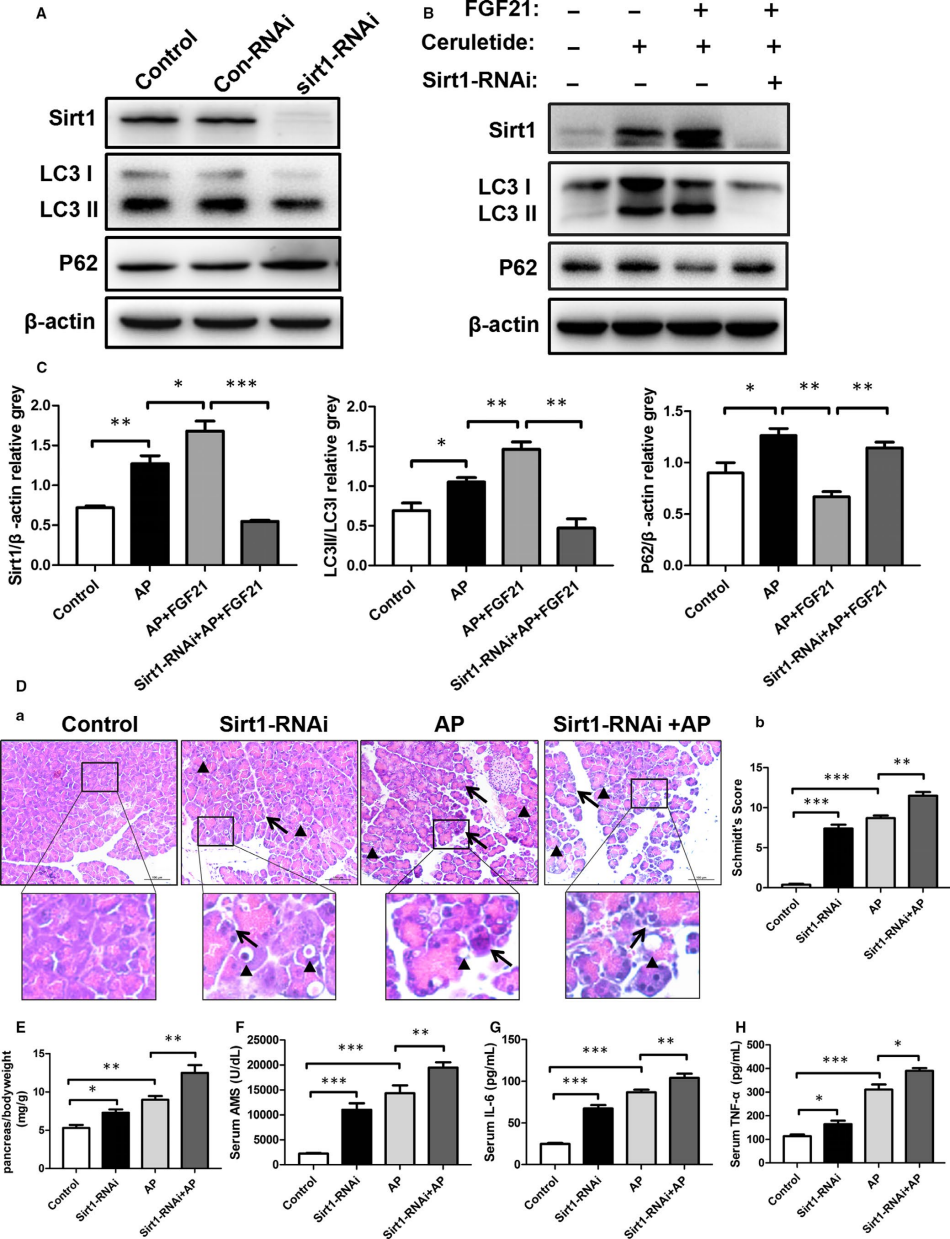
Loss of Sirt1 inhibits activation of autophagy and accelerates cerulein‐induced acute pancreatic injury. A, Expression levels of Sirt1, LC3II/I, Beclin1 and P62 cells after interference of Sirt1‐RNAi in pancreatic tissue. B, C, Expression levels of Sirt1, LC3II/I and P62 in pancreatic tissue of various groups determined by Western blot. D (a), H&E staining of pancreatic tissue (black triangles: the tissue vacuoles, black arrows: the necrosis of the cell). (b) Schmidt's scores of pancreatic tissue. E, Changes of wet weight of pancreas in mice in each group. F‐H, The changes of serum AMS, IL‐6 and TNF‐α expression in mice of each group. ***P < .001, **P < .01, *P < .05

To further investigate the role of Sirt1 in cerulein‐induced AP, we next evaluated whether blockage of Sirt1 would accelerate cerulein‐induced AP in mice. As expected, inhibition of Sirt1 by transfecting with Sirt1‐RNAi largely precipitated cerulein‐induced acute pancreatic injury in mice and markedly worsened the impairment of cerulein‐induced autophagy (Figure 6A‐D). Surprisedly, the protective effect of FGF21 in response to cerulein‐induced AP was also prominently weakened in Sirt1‐RNAi‐treated mice (Figure 6E,F), suggesting that the protective effect of FGF21 against acute pancreatic injury by cerulein may be mediated by Sirt1.

We further evaluated whether Sirt1 plays an important role in the protective effect of FGF21 against cerulein‐induced pancreatic injury. As expected, inhibition of Sirt1 by transfection with Sirt1‐RNAi significantly blocked FGF21 and cerulein‐activated autophagy and largely restrained FGF21‐caused protective effect against cerulein‐induced pancreatic cellular injury as well as the production of inflammatory factors (Figure S3). Taken together, Sirt1 mediated autophagy activation presents an important role of FGF21 against cerulein‐induced AP.

## 4 DISCUSSION

In this study, we found that expression of FGF21 was up‐regulated after the onset of AP. We also found that FGF21 alleviated the pathological damage of AP by promoting expression of Sirt1, repairing damaged mitochondria and lysosomes and alleviating autophagy abnormalities, thereby inhibiting abnormal expression of digestive enzymes and the inflammatory reaction (Figure S4).

Our study found that the expression of FGF21 increased to a peak at 4 hours after AP onset in the mouse model and then decreased gradually. In vivo experiments, FGF21 peaked 2 hours after stimulation with cerulein, suggesting that FGF21 expression increase is a rapid process in the development of AP. This is supported by the presence of serum FGF21 in patients with severe and mild AP. The growth rate of serum FGF21 in mild AP patients was significantly greater than those in severe AP patients. Therefore, we speculate that FGF21 may play a protective role in the early stage of AP, and with the deterioration of the disease, pancreatic cells were damaged, resulting in a decrease in the expression of FGF21.

The expression of serum FGF21 and Sirt1 in patients with AP both increased, and the trend of their expression was consistent. The increase of serum FGF21 in patients with severe AP was weaker than that of patients with mild AP, and the expression of Sirt1 was not substantial, suggesting that FGF21 may have a potential regulatory effect on Sirt1 in patients with AP. Our animal and cell models also showed that the expression of Sirt1 in AR42J cells increased after stimulation with different concentrations of FGF21, also suggesting that FGF21 may regulate the expression of Sirt1 in AP.

In the present study, we determined whether the protective effect of FGF21 on AP is related to autophagy. In AP, intracellular autophagosome numbers increased significantly, most mitochondrial ridges disappeared, the margins of the matrix dissolved and faded out, and expression levels of LAMP1 and LAMP2 in cells were also significantly lower. Therefore, we believe that AP is characterized by damage to mitochondria, lysosomes and abnormal autophagy function.

LAMP1 and LAMP2 are two key proteins that mediate the integration between autophagosomes and lysosomes in autophagy. The integration of autophagosomes and lysosomes was found to be severely impaired in LAMP2‐knockdown mice.^26^, ^27^ The integrity of mitochondrial function is the basis of cell survival. Mitochondrial dysfunction can lead to cell dysfunction, manifested as disease or even death. Mitochondrial damage in AP may be one of the reasons of pancreatic acinar cell death.^28^, ^29^ AP can cause serious disturbances in key organelles, lysosomes and mitochondria.^30^, ^31^ Lysosomal dysfunction plays an important role in the pathobiology of pancreatitis. Impaired autophagy mediates the accumulation of vacuoles in acinar cells. Mitochondrial damage increases the demand for efficient lysosome degradation by stimulating autophagy. Nevertheless, lysosome damage leads to the accumulation of a large number of autophagosomes that fail to integrate with lysosomes, thereby exacerbating the pathological consequences of lysosome dysfunction.^32^ Autophagy is activated in AP, manifested as increased autophagosome numbers. However, the process is functionally impaired, as evidenced by its inefficient progress/dissolution (throughput) because of the deficiency of lysosome and mitochondria function. In the present study, we showed that damage of mitochondria and lysosomes in pancreatic cells of AP was significantly reduced after treatment with FGF21, and number of autolysosomes increased, suggesting that FGF21 might mediate repair of mitochondria and lysosomal function, promoting lysosome‐induced degradation of autophagosomes and alleviating the damage to autophagy.

It has been reported that Sirt1 activates autophagy and increases the clearance of damaged cells by autophagy.^33^ In the present study, we found that FGF21 regulated the expression of Sirt1 and repaired autophagic dysfunction after the onset of AP. When lentivirus harbouring Sirt1‐RNAi was used to inhibit the expression of Sirt1 in pancreatic tissue in mice, the alleviating effect of FGF21 on AP weakened, and the regulatory effects of FGF21 and autophagy were counteracted, suggesting that Sirt1 plays an important role in the alleviation of AP mediated by FGF21. Therefore, we suggest that FGF21 alleviates the effects of AP by numerous mechanisms, including promotion of expression of Sirt1, alleviating the damage to mitochondria and lysosomes, repairing abnormal autophagy and inhibiting the abnormal expression of digestive enzymes and inflammatory reaction. Xiao et al proposed that spautin‐1 alleviated AP by effectively inhibiting autophagic flow.^34^, ^35^ Zhang et al also reported that the antioxidant, astaxanthin, alleviated damage caused by AP via regulation of the JAK/STAT3 pathway and inhibition of autophagic flow,^36^ suggesting that the effects of AP could be reduced by inhibiting autophagic flow. In our study, FGF21 effectively alleviated the autophagic obstruction caused by AP by repairing mitochondria and damaged lysosomes, suggesting that alleviation of autophagic obstruction in AP plays an important role in the treatment of AP.

## CONFLICT OF INTEREST

The authors declare no conflict of interest.

## Supporting information

Fig S1

Fig S2

Fig S3

Fig S4

## Data Availability

The data that support the findings of this study are available from the corresponding author upon reasonable request.
