# Supplementary figures and images for: Fibroblast growth factor 21 alleviates acute pancreatitis via activation of the Sirt1‐autophagy signalling pathway
Source: J Cell Mol Med. 2020 Mar 31;24(9):5341–51. doi: 10.1111/jcmm.15190 (PMC7205819; doi:10.1111/jcmm.15190)

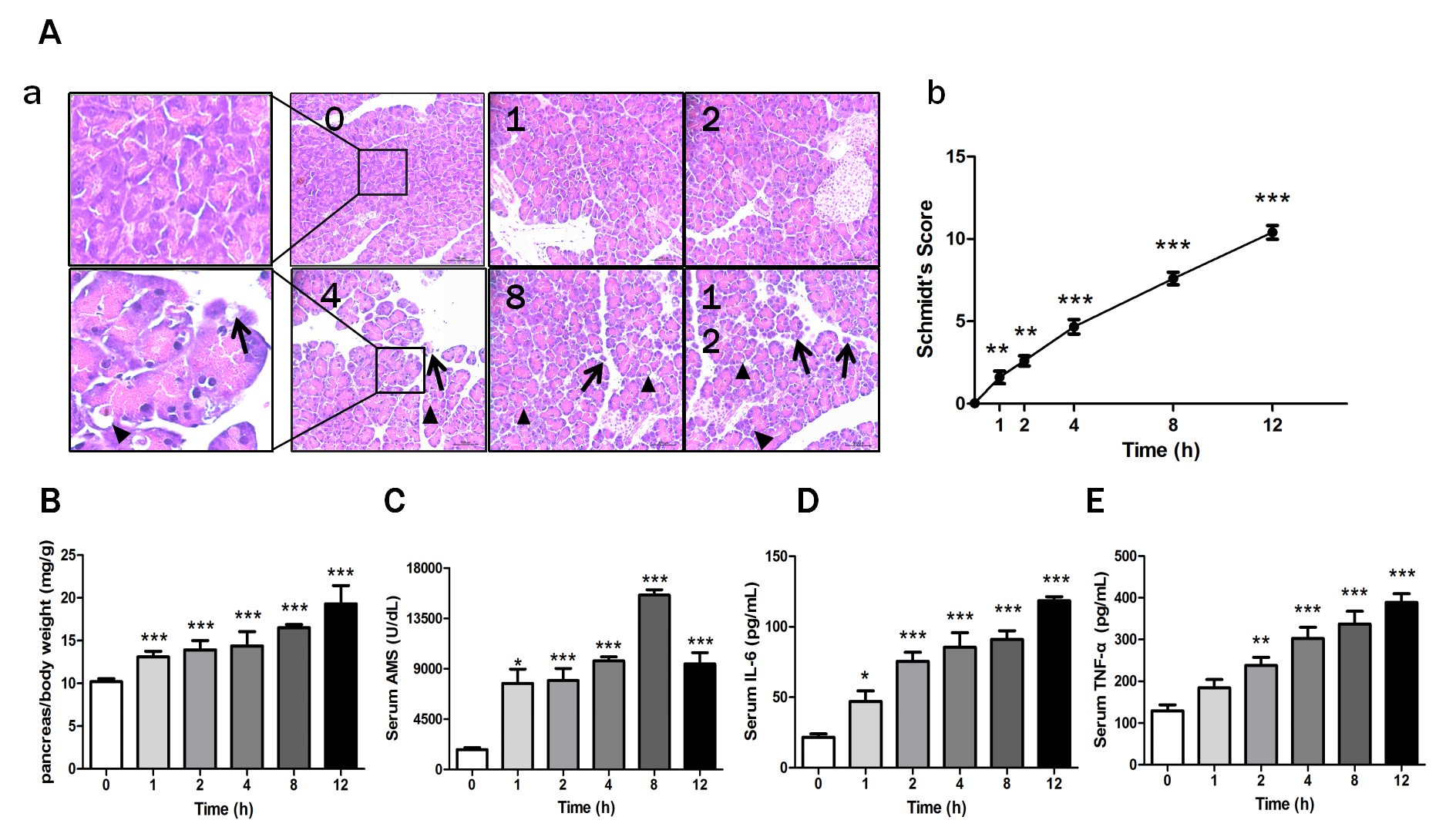

Supplement: Supplementary file 1 — Fig S1 [file JCMM-24-5341-s001.tif]

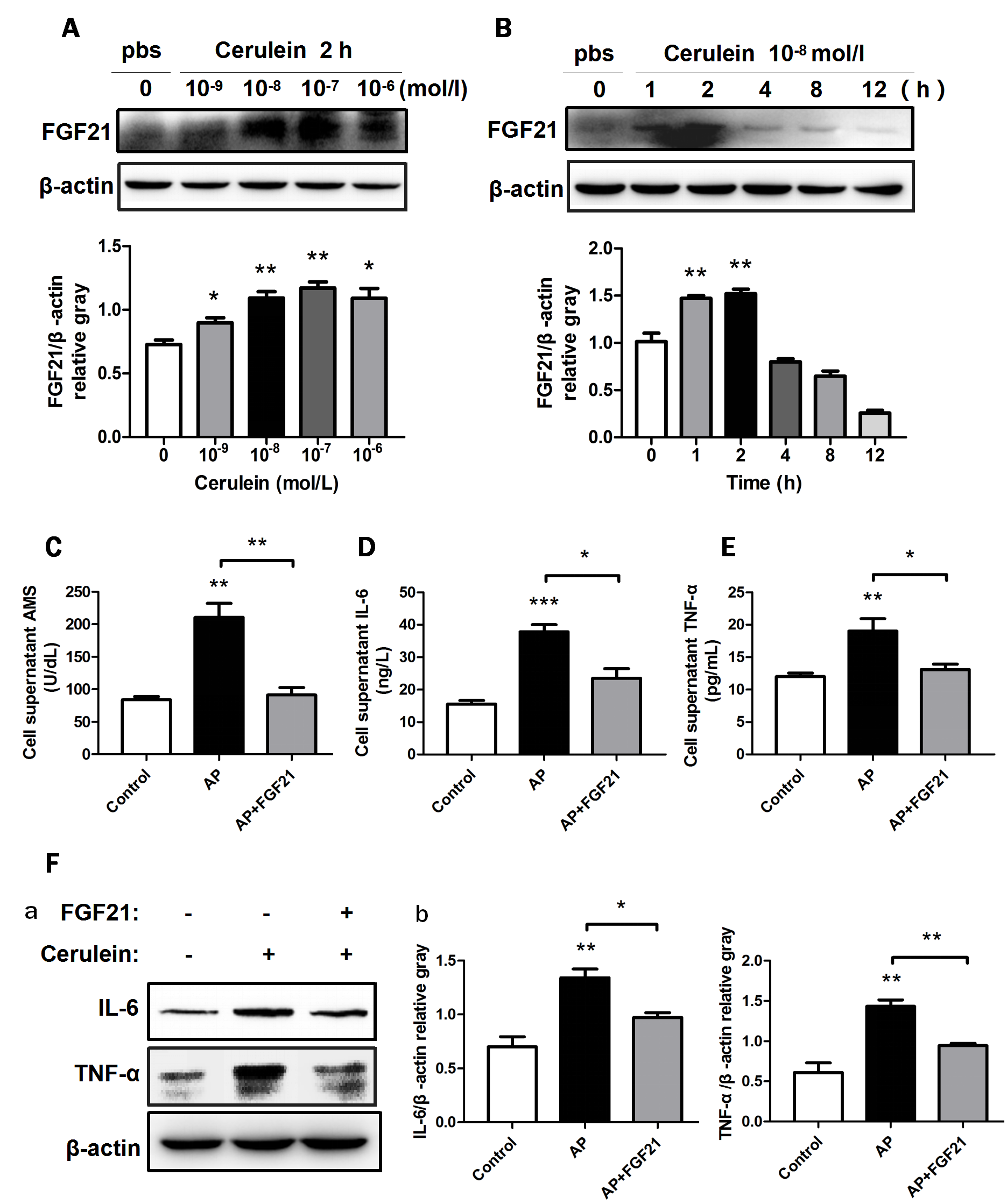

Supplement: Supplementary file 2 — Fig S2 [file JCMM-24-5341-s002.tif]

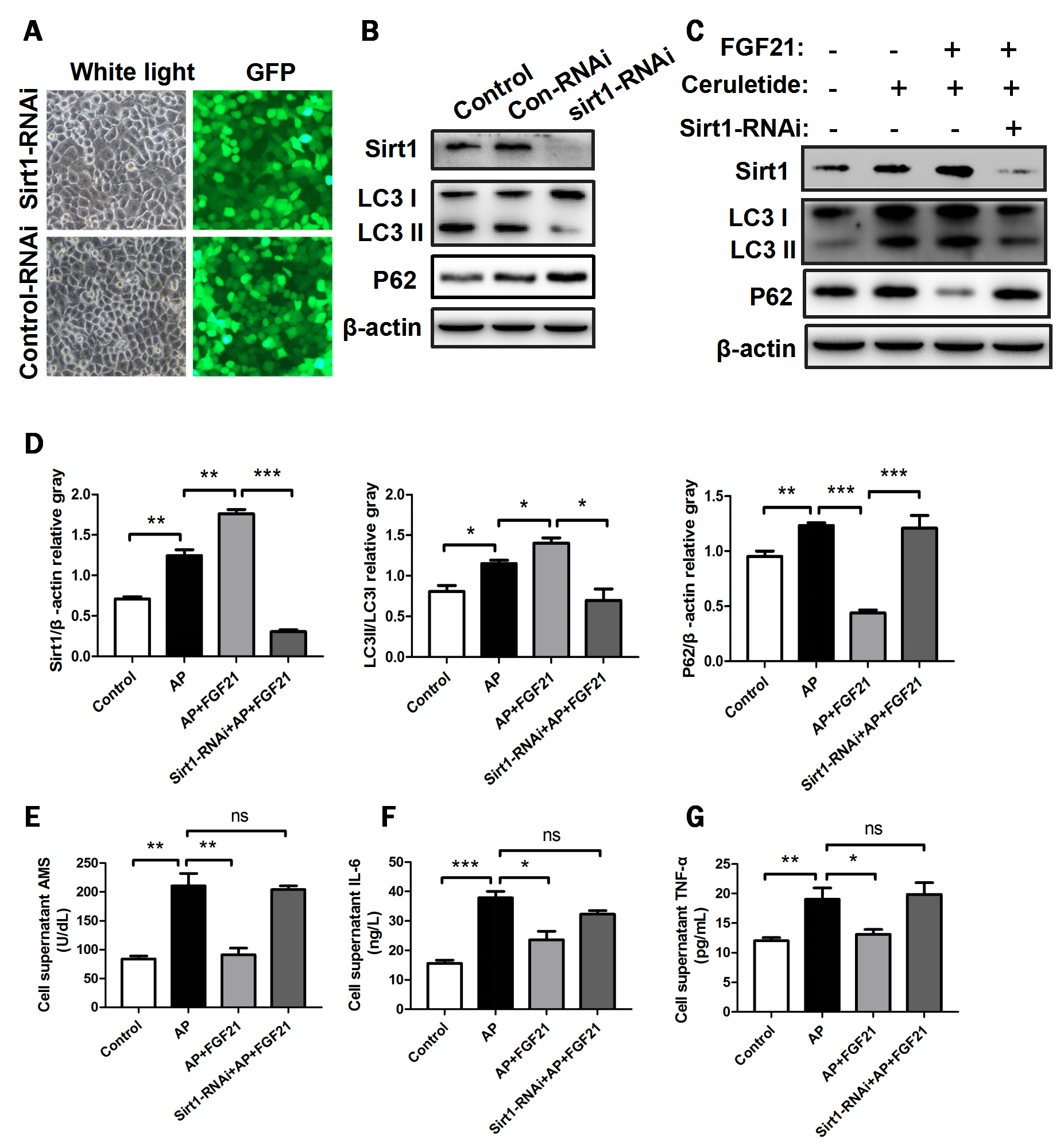

Supplement: Supplementary file 3 — Fig S3 [file JCMM-24-5341-s003.tif]

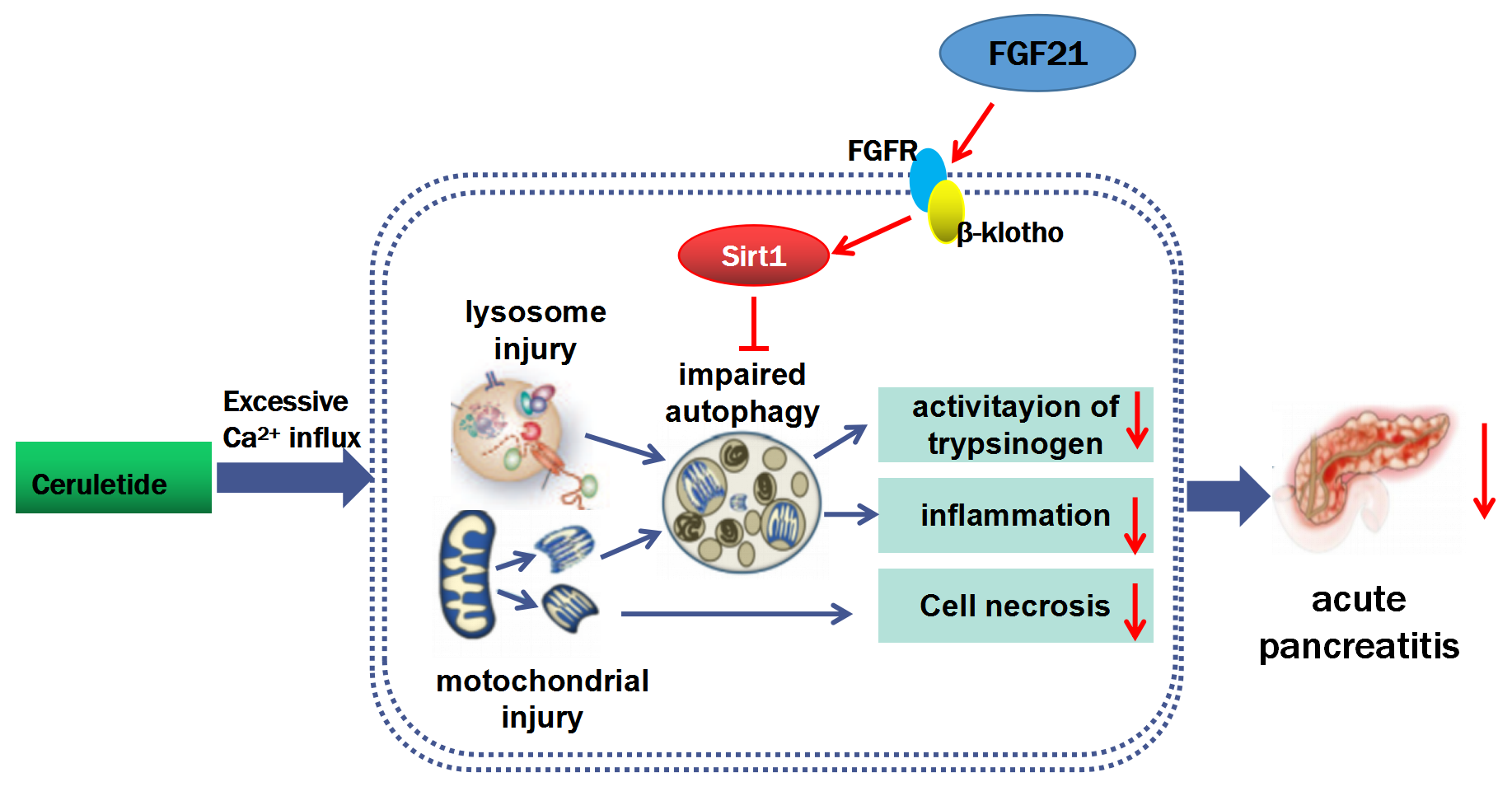

Supplement: Supplementary file 4 — Fig S4 [file JCMM-24-5341-s004.tif]
